# *Poa laegaardiana*, a new species from Ecuador (Poaceae, Pooideae, Poeae, Poinae)

**DOI:** 10.3897/phytokeys.100.25387

**Published:** 2018-06-21

**Authors:** Paul M. Peterson, Robert J. Soreng

**Affiliations:** 1 Department of Botany MRC-166, National Museum of Natural History, Smithsonian Institution, Washington, DC 20013-7012, USA

**Keywords:** Ecuador, *Poa*, Poaceae, taxonomy

## Abstract

*Poa laegaardiana* **sp. nov.**, is described and illustrated. The new species was found growing on sandy, volcanic soil in *Festuca*-*Calamagrostis* dominated grasslands southwest of Ambato and 2.2 km from Fecundo Vela in Provincia de Bolivar. The new species is morphologically similar to *Poa gigantea* but differs in having glumes 3/4 to 7/8 as long as the adjacent lemmas, a callus with a sparse, short, dorsal tuft of woolly hairs, culms 50–72 cm tall and spikelets 4.1–4.8 mm long. In addition, we include a key to the narrow-spikelike panicled species of *Poa* in Ecuador.

## Abstract (es)

Se describe e ilustra una nueva especie, *Poa laegaardiana* **sp. nov.** La especie se encontró creciendo en suelo arenoso y volcánico en pastizales dominados por *Festuca*-*Calamagrostis* al suroeste de Ambato y a 2.2 km de Fecundo Vela en la Provincia de Bolívar. La nueva especie es morfológicamente similar a *Poa gigantea*, pero se distinque por glumas ¾ a 7/8 tan larga como los lemmas adyacentes, un callo con un mechón escaso, corto, dorsal de pelos lanosos, culmos de 50–72 cm de altura, y espiguillas 4.1–4.8 mm de largo. Además, incluimos una clave para las especies de *Poa* con panículas espiciformes de Ecuador.

## Introduction

*Poa* L., one of the two largest genera of grasses, is distributed in temperate regions of both hemispheres and in mountainous regions of the tropics (Soreng et al. 2017). Hitchcock (1927) in his treatment of the grasses of Ecuador, Peru and Bolivia reported eight species of *Poa* occurring in Ecuador. Hjorth (1991) prepared descriptions and a key to 11 species of *Poa* in Ecuador. Jørgensen and León-Yánez (1999) listed 14 species of *Poa* in Ecuador. Of names not in synonymy in the latter checklist, we consider *Poa kunthii* Lindm., nom. nov. for *Poa remota* Kunth, non Forselles, a synonym of *Lolium arundinaceum* (Schreb.) Darbysh.; *Poa pinchachensis* Hack., nom. nov. for *Poa trachyphylla* Hack., non Pilg., a synonym of *Poa trivialis* L. and; *Poa paramoensis* Laegaard a synonym of *Poa huancavelicae* Tovar (Sylvester et al. 2016). In addition, *Aphanelytrum procumbens* Hack. is now treated as *Poa hitchcockiana* Soreng & P.M. Peterson (Peterson and Soreng 2016). This brings the total to 16 known species of *Poa* in Ecuador including our new one.

The subtribe Poinae Dumort. is a large assemblage of 550 species represented by a single, monophyletic genus, *Poa* (Gillespie et al. 2007, 2008; Soreng 1990; Soreng et al. 2017). Species within *Poa* are morphologically highly variable and are characterised by having monoclinous or diclinous flowers, flag leaf sheath margins fused 1/15–3/4 (or more) from the base, leaf blades usually with an adaxial groove on each side of the midvein, ligules hyaline to sub-chartaceous, paniclulate inflorescences, spikelets that are usually laterally compressed with 2–6 (rarely 1 or more than 6) florets that disarticulate individually above the glumes, keeled glumes usually with 1–3 veins and usually shorter than the lowest lemma, callus glabrous or webbed with soft woolly hairs (rarely a crown of hairs), lemmas 3–5(–11)-veined, usually keeled (apex rarely with a brief awn), 2 lodicules, each with a lateral lobe, glabrous ovaries, caryopses elliptical to fusiform, short hilum, endosperm hard with lipid and a base chromosome number of *x* = 7 (Soreng 2007; Soreng and Peterson 2012; Zhu et al. 2006). Based on molecular phylogenetic studies, *Poa* has been divided into five subgenera: *Sylvestres* (V.L. Marsh ex Soreng) Soreng & L.J. Gillespie, *Ochlopoa* (Asch. & Graebn.) Hyl., *Pseudopoa* (K. Koch) Stapf, *Stenopoa* (Dumort.) Soreng & L.J. Gillespie and *Poa*, corresponding to the five major clades (Gillespie et al. 2007, 2008; Giussani et al. 2016; Soreng et al. 2010).

While reviewing Ecuadorian specimens of *Poa*, RJS found an interesting undetermined specimen collected in 1990 by PMP and Carol R. Annable (*Peterson 8997 & Annable*) from the Provincia de Bolivar southwest of Ambato and 2.2 km from Fecundo Vela. The specimen has unique morphological characters but superficially resembles *Poa gigantea* (Tovar) Refulio, known only from Peru and *P. subspicata* (J. Presl) Kunth, known from Columbia, Ecuador, Peru and Venezuela. We describe it as a new species of *Poa* and, to aid in identification, include a key to all narrow and spikelike-panicled species of *Poa* in Ecuador.

## Taxonomy

### Poa laegaardiana

Taxon classificationPlantaePoalesPoaceae

Soreng & P.M. Peterson sp. nov.

urn:lsid:ipni.org:names:60476587-2

Fig. 1A–J

#### Type.

Ecuador. Province de Bolivar, 66.5 km SW of Ambato on hwy to Guaranda and 2.2 km along road to Facundo Vela, [est. 1.516022 S, 79.007192 W], 4300 m a.s.l., growing on sandy, volcanic soil in *Festuca*-*Calamagrostis* [s.l.] grassland, 3 May 1990, *P.M. Peterson 8997 & C.R. Annable* (holotype: US-3244349!; isotypes: AAU!, MO-3853338!, QCA!).

#### Diagnosis.

Differing from *Poa gigantea* (Tovar) Refulio in having glumes 3/4 to 7/8 as long as the adjacent lemmas (versus glumes about as long or longer than the adjacent lemma), a callus with a sparse, short, dorsal tuft of woolly hairs (versus no web), culms 50–72 cm tall (versus 22–58 cm tall) and spikelets 4.1–4.8 mm long (versus 5–5.5 mm long).

**Figure 1.**
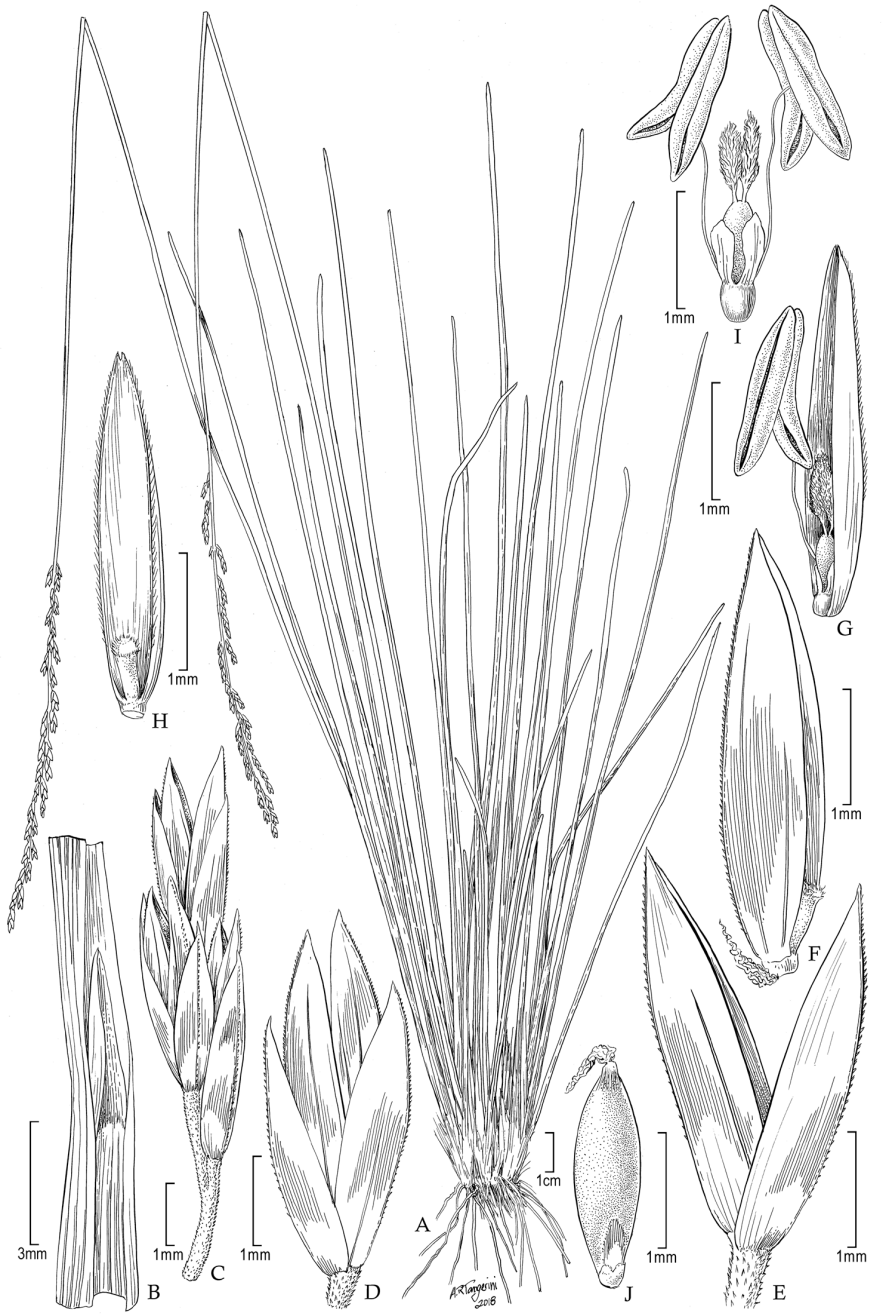
*Poa laegaardiana*: **A** Habit **B** Sheath, ligule and blade **C** Panicle branch **D** Spikelet **E** Glumes **F** Floret **G** Palea enclosing lodicules, ovary and stamen **H** Palea, ventral view **I** Lodicules, ovary and stamens **J** Caryopsis. Drawn from the holotype collection (*Peterson 8997 & Annable*).

#### Description.

Gynomonoecious. **Perennials**, densely tufted, without lateral tending shoots or with infrequent short lateral tending shoots, greyish-green, with anthocyanic tinges; tillers extravaginal, erect; **culms** 50–72 cm tall, erect, culm nodes and internodes, terete, smooth, glabrous, 0 nodes exposed, highest node in lower 1/10 of culm. **Leaves** concentrated at base; **sheaths** 14–30 cm long, laterally compressed, slightly keeled, smooth, glabrous, proximal sheaths chartaceous, strongly overlapping at base, butt sheaths papery, smooth, glabrous and inconspicuously shredding with age; **uppermost culm sheath** to 30 cm long, margins fused ca. 10% their length, ca. 3–4× longer than their blade; **throats and collars**, smooth, glabrous; **ligules** to 3.5–6.6 mm long, of sterile shoots to 3.5 mm long, membranous, abaxially scaberulous, margins decurrent, apicies acute, apex of distal-most sometimes fimbriate; **blades** to 22 cm long, 1.5--3 mm wide (expanded), uppermost to 8 cm long, firm, stiff, erect, tightly folded to involute, keeled, abaxially smooth or obscurely scaberulous along the veins, adaxially densely scabrous, mostly along the veins on a nearly level surface (aside from the single channels flanking the central vein), apex narrowly prow tipped, acuminate, stiff; **sterile shoot blades** like those of the culm, crowded, to 22 cm long above the initial cataphylls if any. **Panicles** 7.6–11.5 cm long, 0.6–1.2 cm wide, contracted, spiciform, erect, narrow, interrupted below with areas along the rachis with no branches, with 50–60 spikelets; **proximal internodes** 2.5–3.5 cm long, smooth, axis with mostly 2 branches at lower nodes; **branches** 2.5–3.5 cm long, with up to 12 spikelets crowded in the distal 2/3, appressed (ascending at anthesis), terete to slightly angled, short scabrous mainly along the angles; **lateral pedicels** mostly 0.5–1 mm long. **Spikelets** 4.1–4.8 mm long, 2–2.5× longer than wide, lanceolate, laterally compressed, not bulbiferous, violaceous in part at maturity, florets 2, the upper slightly reduced; **rachilla** internode above the proximal floret 0.5 mm long, terete, scabrous to hirtellous; **glumes** 3/4 to 7/8 as long as the adjacent lemma, subequal, lanceolate to oblong, keels smooth or distally obscurely scaberulous, margins distally smooth, apex acute; **lower glumes** 3–3.5 mm long, 1 (3)-veined; **upper glumes** 3.4–3.8 mm long, 3-veined; **calluses** webbed, with a sparse, short, dorsal tuft of woolly hairs to about 1 mm long; **lemmas** 3.5–4.2 mm long, 5-veined, lanceolate in side-view, violaceous in part, strongly laterally compressed, distinctly keeled, glabrous throughout, keel distally scaberulous or nearly smooth, sides smooth (appearing densely granular due to abundant short-cells), intermediate veins obscure to distinct, margins inrolled below at maturity, narrowly scareous above, edges smooth, apicies acute; **paleas** 3.3–3.7 mm long, a little shorter than the lemma, texture like the lemma, 2-keeled, the keels distally scaberulous, glabrous between the keels. **Flowers** pistillate over perfect within the spikelets; **lodicules** 0.25 mm long, 2, lobed; **stamens** 3, **anthers** 1.4–1.6 mm long, light yellow, vestigial in upper floret less than 0.1 mm long; **ovary** glabrous; **caryopses** 1.6–2 mm long, elliptical in side-view, brown, translucent, sulcus broad and shallow, hilum ca. 0.15 mm long, round, grain loosely adherent to the palea.

#### Distribution.

The species is known only from the type collection in Provincia Bolivar, Ecuador.

#### Conservation status.

The species is apparently rare. Google Earth view [26 Mar 2018] of the pass location where the new species was collected in 1990 indicates the area is now covered by small farms.

#### Etymology.

The specific epithet honors Simon Laegaard (1933–), a renowned Danish Botanist, who has made extensive collections in Ecuador, Greenland and South America.

#### Discussion.

There are a number gynomonoecious species of *Poa* occurring in northern South America, Central America and central Mexico but all have loose, open panicles, except the new species and species of Poa sect. Dissanthelium (Trin.) Refulio (Refulio-Rodriguez et al. 2012). Most of these open-panicled species of Poa supersect. Homalopoa (Dumort.) Soreng & L.J. Gillespie and the new species have glabrous lemmas and a web on the callus with a perfect lower floret and a pistillate upper floret. The new species appears to belong within Poa subg. Poa supersect. Homalopoa (Giussani et al. 2016). Poa sect. Homalopoa s.str. may be restricted to Eurasia and North America while most species of the New World are placed in sect. Homalopoa s.l. (Giussani et al. 2016; Soreng et al. 2003).

*Poa gigantea* (Poa sect. Dissanthelium) is morphologically similar to our new species but differs in having glumes longer than or equalling the adjacent lemma, an unwebbed callus, shorter culms 22–58 cm tall and larger spikelets 5–5.5 mm long (Tovar Serpa 1985; Refulio-Rodriguez 2012). Other Ecuadorian species of *Poa* with narrow, spikelike panicles includes: *P. chamaeclinos* Pilg., *P. scaberula* Hook. f. and *P. subspicata*. We provide a key to separate these from the new species below (Hjorth 1991; Soreng and Peterson 2012).

### Key to the narrow, spikelike-panicled species of *Poa* in Ecuador

**Table:** 

1	Panicles 0.8–1.5 cm long; culms 1–3.5 (–5) cm tall; glumes obovate to sub-flabellate; callus glabrous	***P. chamaeclinos***
–	Panicles 2.5–18(–20) cm long; culms (5–) 8–75 cm tall; glumes lanceolate to oblong; callus with a web of woolly hairs	**2**
2	Spikelets (2.5–) 3–4 mm long; anthers 0.3–0.7 mm long; short-lived perennials	***P. scaberula***
–	Spikelets (3.5–) 4–6.5 mm long; anthers 0.5–1.6 mm long; long-lived perennials	**3**
3	Glumes 3/4 to 7/8 as long as the adjacent lemma; anthers 1.4–1.6 mm long; spikelets with 2 florets; lemma glabrous	***P. laegaardiana***
–	Glumes 1/2 to 3/4 as long as the adjacent lemma; anthers 0.5–1.2 mm long; spikelets with 3, sometimes 4 florets; lemma keels and marginal veins sparely to densely sericeous to villous basally to near apex or infrequently glabrous throughout	***P. subspicata***

## Supplementary Material

XML Treatment for Poa laegaardiana
